# Botulinum toxin: An emerging therapy in female bladder outlet obstruction

**DOI:** 10.4103/0970-1591.56181

**Published:** 2009

**Authors:** Aditya A. Pradhan

**Affiliations:** Department of Urology, Military Hospital, Cantonment, Jalandhar, Punjab 144 005, India

**Keywords:** Botulinum toxin, dysfunctional female voiding

## Abstract

**Introduction:**

We evaluated the utility of botulinum toxin in functional female bladder outlet obstruction.

**Materials and Methods:**

A total of 7 consecutive female patients with bladder outlet obstruction were included. Patients with neurogenic bladder were excluded. All were previously treated with periodic dilations. Diagnosis was based on symptomatology, cystometry, and micturating cystograms. A total of 2 patients had been in chronic retention with residual volumes more than 400 ml. All were managed with an injection of botulinum toxin, 100 units in 2 ml of saline injected with a flexible cystoscopic needle. The site of the injection was deep submucosally, 0.5 ml in each quadrant at the level of the most prominent narrowing seen endoscopically. All the procedures were done as day care procedures under local anaesthesia. After the procedure, no catheter was placed. Patients were followed up for changes in IPSS scores and post void residual urine measurements. In all cases, multiple injections were used.

**Results:**

The follow-up period ranged from 48–52 weeks. The IPSS reduced by an average of 12 points. Post void residual urine reduced by 62%. Improvements commenced 4.85 days (average) after the procedure and lasted for an average of 16.8 weeks (range: 10.8–28 weeks).

**Discussion:**

There is a gradual improvement in symptoms over time and the maximal effect occurred at 10–14 days. The duration of improvement was approximately 16.8 weeks. All patients were satisfied by the degree of improvement felt.

**Conclusions:**

Botulinum toxin proved successful in improving the voiding characteristics. It possibly acts at the zone of hypertonicity at the bladder neck or midurethra. The only disadvantage is the high cost of the drug.

## INTRODUCTION

Bladder outlet obstruction in females remains a poorly defined entity with many definitions but no universally accepted diagnostic criteria. Nitti and Patel stated that it is best diagnosed by a combination of history, physical examination, and videourodynamic studies.[1] Treatment options are limited including periodic dilations, bladder neck incision, sacral neuromodulation or, more recently, open urethroplasty.[2,3] Botulinum toxin has also recently been tried in this condition with some success.[4]

In our practice, we found a high level of dissatisfaction with the commonly practiced technique of periodic dilation to treat this condition. Saccral neuromodulation is not available in our center due to cost constraints. Hence, we decided to see the efficacy of botulinum toxin in this condition.

## MATERIALS AND METHODS

**Figure 1:**
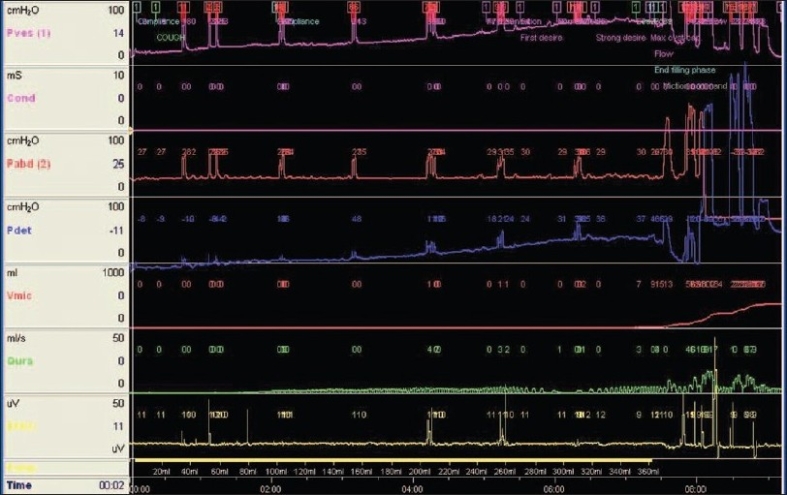
A pressure flow study of female bladder outlet obstruction

**Figure 2:**
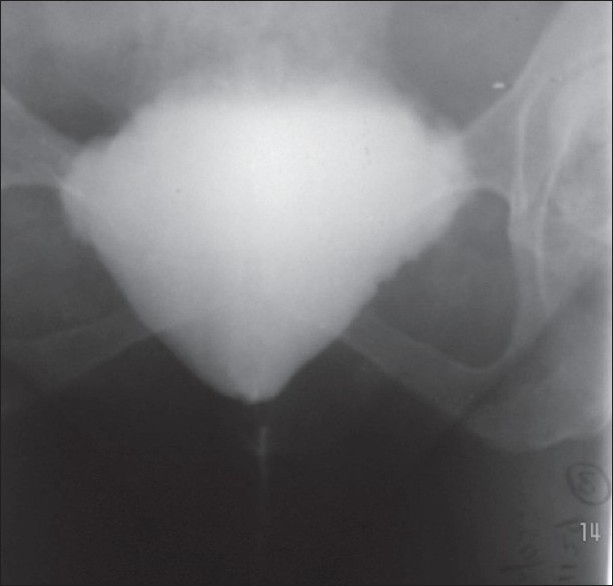
Micturating cystogram showing bladder outlet obstruction

**Figure 3:**
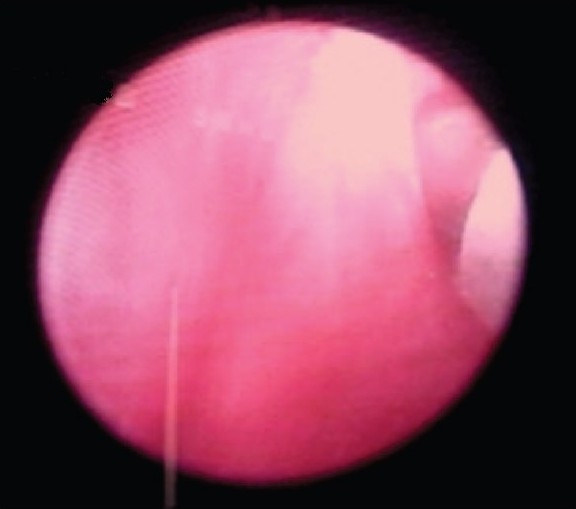
Needle injection at point of maximum narrowing seen on cystoscope

A total of 7 consecutive female patients with bladder outlet obstruction were included in this study. Patients with neurogenic bladders and traumatic strictures were excluded. All were previously treated with periodic dilations and highly dissatisfied with the outcome. The diagnosis of bladder outlet obstruction was based on symptoms combined with pressure flow studies [Figure 1], micturating cystourethrograms [Figure 2], and cystoscopy [Figure 3]. Video urodynamic facility was not available at our center. A total of 2 patients had been in chronic retention with residual volumes more than 400 ml.

All patients were managed with an injection of botulinum toxin, 100 units in 2 ml of saline injected with a flexible cystoscopy needle. In all cases, the preparation was botulinum toxin Type A, 100 units per vial manufactured by Allergan. The cystoscopy needle used was the standard flexible 22 gauge William's cystosopy needle manufactured by Cook Medical. The injection was done deep submucosally, 0.5 ml in each quadrant at the level of most prominent narrowing seen endoscopically. All procedures were day care procedures done under local anesthesia, using 2% Lignocaine Jelly applied topically. After the procedure, no catheter was placed. Patients were followed up for changes in IPSS scores and post void residual urine measurements. Reinjection was done when the patient returned to preinjection symptom status. In all cases, multiple injections were used: 4 injections for 1 patient, 3 injections for 3 patients, and 2 injections for the remaining cases.

Dilation was used post procedure after 2 weeks for 1 patient. The International Prostate Symptom Score (IPSS) was recorded post procedure after 2 weeks of treatment and monthly thereafter. Post-void residual urine was recorded after 2 weeks of treatment. The endpoints chosen were change in status of symptoms and post-void residual urine volume.

## RESULTS

The follow-up period ranged from 48–52 weeks. There was a reduction in IPSS by an average of 12 points [Table 1]. Post-void residual urine was reduced by an average of 62%. Improvements commenced 3–12 days after the procedure with an average of 4.85 days. The duration of improvement lasted for 10.8–28 weeks with an average of 16.8 weeks.

**Table 1:** Patient details

Patient no.	IPSS before and after treatment the difference in brackets		QMax	P Det at Qmax	Post void residual urine before and after treatment		Onset of improvement (days)	Duration of improvement (weeks)
						
	Pre	Post			Pre	Post		
1	23	10 (13)	07	56	230	76	4	17.2
2	25	15 (10)	12	53	560	190	12	14.4
3	24	6 (18)	08	43	240	90	3	28
4	19	7 (12)	14	35	110	34	5	20.8
5	23	12 (11)	09	75	480	170	3	10.8
6	17	7 (10)	10	32	125	45	3	12.4
7	19	9 (10)	11	28	98	36	4	14

IPSS= International Prostate Symptom Score. Q Max = Max flow rate in ml/s Pdet at Qmax = Detrussor Pressure at Q max. Post void Residual Urine measured in ml, by abdominal ultrasound								

In most patients, the subjective improvement started after the third day after the injection. In 1 case, there was no improvement until the 12^th^ day and the patient continued to have severe obstructive symptoms. Hence, one dilation to 20 fr was done. Thereafter, she showed improvement in symptoms, which was sustained for almost 14 weeks. On an average, patients remained symptom-free for 16.8 weeks after an injection.

## DISCUSSION

Botulinum toxin is derived from the anaerobic spore bearing bacillus Clostridium botulinum. It is one of the most toxic substances known to man with the potential to kill 1 million humans with 1 gm of the pure toxin. Botulinum toxin acts at the myoneural junction on the acetylcholine receptors. It produces an irreversible blockade, which usually lasts about 3 m. Its medical applications were first investigated for management of strabismus by Schantz and Scott in 1968. In 1989, the FDA approved the marketing of botulinum toxin in the USA for the treatment of blepharospasm and strabismus in patients 12 years of age and above. Since then its use has spread to various applications in cosmetology and neurology mainly for correction of neuromuscular spasm. There have recently been numerous reports of its applications in urology. All these have been off-label applications. Botulinum has shown utility in the management of refractory overactive bladder, detrussor sphincter dyssynergia, recurrent urethral strictures, and more recently in Fowlers syndrome.[4,5]

Bladder outlet obstruction in females is multi-factorial. Currently, the diagnosis is made in 2–22% of women with persistent lower urinary tract symptoms (LUTS) who are assessed by urodynamic studies.[1,6] The treatment approaches to date have been periodic dilation, bladder neck incisions, or surgical repair. The British group of Fowler introduced the concept of a primary disorder of sphincter relaxation. In their experience, this is the most common cause of functional bladder outlet obstruction. Of 247 women who presented with retention or voiding dysfunction, 58% were diagnosed with such a condition.[7] This was demonstrated by an elevated urethral pressure profile and increased sphincter volume or by an abnormal electromyogram (EMG) with complex repetitive discharges and decelerating bursts. They reported their experience with saccral neuromodulation and found normal voiding restored in 77% of the cases.[8]

Chancelor's group has reported the use of botulinum toxin in female bladder outlet obstruction. They found marked improvement in daytime and nocturnal voiding symptoms. Two cases developed new onset mild stress incontinence. They used a dose of 100–200 units in 4 ml saline.[4] Hann-Chorng Kuo reported his experience in this disorder in 20 cases of dysfunctional voiding or neurogenic causes with hypocontractile bladders. Patients included 16 females and 4 males. He used 50 units of botulinum toxin. A total of 18 patients (90%) were successfully managed with a reduction in post-void residual volume, urethral closure pressure, and voiding pressures.[9] The mechanism of action of botulinum toxin in this particular application is to reduce the guarding reflex that serves as an inhibitor to bladder contraction.

Patients need to be counseled about the deferred onset of action. It takes 3-4 days before the onset of action, and this does create anxiety in the minds of both the surgeon and patient. We found that there is a gradual improvement in symptoms over time and the maximal effect occurred 10 to 14 days after the injection. The duration of improvement ranged from 10.8 weeks to 28 weeks, longer than the expected duration of action. This phenomenon has also been noted by others. All patients were satisfied by the degree of improvement.

There was a reduction in IPSS by an average of 12 points. Post-void residual urine reduced by average of 62%. The mechanism of action of botulinum toxin in this condition of bladder hypocontractility has previously been studied and speculated to be a reduction in the guarding reflex, which serves as an inhibitor to bladder contraction.[9] The only disadvantage is the cost of therapy amounting to approximately Rs 13,000 per vial of 100 units. The action is reversible and injections need to be repeated every 3-4 months. However, the procedure is a simple day care procedure done under local anesthesia.

## CONCLUSIONS

Idiopathic female dysfunctional voiding is increasingly being recognized as a difficult and inadequately treated condition.

Botulinum toxin has proved successful in improving the voiding characteristics. It possibly acts at the zone of hypertonicity at the bladder neck or midurethra. The only disadvantage is the high cost of the drug, which needs to be compared with the cost of other therapies currently available for this condition.

This is admittedly a small study. Further multi-center studies are indicated for assessing its efficacy, dose range, and improvement in injection delivery systems.
